# A novel frameshift mutation in ubiquitin-specific protease 26 gene in a patient with severe oligozoospermia

**DOI:** 10.1042/BSR20191902

**Published:** 2020-04-23

**Authors:** Leilei Li, Qi Xi, Hongguo Zhang, Jia Fei, Yuting Jiang, Linlin Li, Ruizhi Liu, Ruixue Wang, Guirong Zhang

**Affiliations:** 1 Centre for Reproductive Medicine and Prenatal Diagnosis, First Hospital of Jilin University, Changchun, P. R. China; 2 Peking Medriv Academy of Genetics and Reproduction, Peking, P. R. China

**Keywords:** framshift mutation, male infertility, novel mutation, ubiquitin-specific protease 26

## Abstract

Ubiquitin-specific protease 26 (*USP26*) encodes a predicted protein containing his- and cys- domains that are conserved among deubiquitinating enzymes. *USP26* is specifically expressed in testis tissue and is a potential infertility gene. In the present study, we performed genetic testing related to spermatogenesis impairment in a patient with idiopathic severe oligozoospermia to identify the cause. The patient underwent clinical examination and reproductive hormone testing. Genes associated with male infertility, including *USP26*, were assessed by targeted exome sequencing. A novel frameshift mutation, c.2195delT (p.Phe732Serfs*14), was identified in *USP26*. This frameshift mutation was located in residue 732 of *USP26* gene, leading to loss of the conserved deubiquitinating enzyme His-domain and producing a truncated protein of 744 amino acids. Bioinformatics analysis revealed this mutation to be pathogenic. A novel framshift mutation c.2195delT (p.Phe732Serfs*14) in *USP26* gene was reported to be associated with male infertility in a Chinese patient with severe oligozoospermia.

## Introduction

Infertility is a common global disease that affects 15% of couples of childbearing age. Infertility caused by male factors accounts for approximately 50% of all infertility cases [1–4]. More than 50% of the causes of male infertility are still unclear. In recent years, great attention has been focused on genetic factors including AZF microdeletions, chromosomal abnormalities, gene mutations, and gene polymorphisms [5]. Genetic factors account for approximately 15–30% of all male infertility and affect physiological processes including hormone production, spermatogenesis, and sperm quality [6,7].

Many genes on the X chromosome are thought to be involved in male infertility [8]. *Ubiquitin-specific protease 26* (*USP26*), which contains only a single exon, is located at Xq26.2 (Online Mendelian Inheritance in Man (OMIM) 300309). *USP26* contains 2794 base pairs encoding 913 amino acids with a molecular mass of 104 kilodaltons. *USP26* shows testis-specific expression. In mouse testes, *USP26* is highly expressed in sperm cells, sperm round cells, and the blood–testis barrier [9]. In humans, *USP26* colocalizes with the androgen receptor (AR), and is highly expressed in testicular Leydig cells and sertoli cells [10]. *USP26* is X-linked and encodes a deubiquitinating enzyme (DUB) [11]. Due to the important role of DUBs in spermatogenesis, *USP26* is thought to play an important role in male fertility.

Previous reports have identified up to 20 *USP26* polymorphisms, mostly occurring in gene clusters, that may be associated with male infertility [8]. A 370-371insACA, 494T>C, and 1423C>T gene cluster mutation in *USP26* is most commonly reported in infertile male patients [12]. The c.370-371insACA, c.494T>C, c.1423C>T, c.1090C>T, c.1737G>A cluster, with five *USP26* mutations, have no effect on deubiquitinating enzyme activity, and enzyme activity and meta-analyses revealed that there is no direct correlation between *USP26* mutation and male infertility [13]. It is clear that the relationship between *USP26* and male infertility remains controversial and needs to be fully elucidated. Only a small fraction of *USP26* mutations are thought to cause pathogenic changes in male fertility. Here, we report a patient with severe oligozoospermia carrying a c.2195delT pathogenic mutation in the *USP26* gene. This is a novel mutation that has not been reported previously. Our results complement the *USP26* mutation spectrum, provide strong etiological evidence, and indicate the need for genetic counseling in male patients with idiopathic infertility.

## Materials and methods

### Patient

The patient was a 30-year-old man who presented with primary infertility having had 5 years of regular unprotected intercourse. He visited our center for his reproductive problem. Female factors were excluded. Two routine semen analyses, performed according to the World Health Organization guidelines [14], revealed severe oligozoospermia. The known causes of male infertility, including obstructive causes, infectious factors, genitourinary injury factors, chromosomal abnormalities, and AZF microdeletions, were excluded. Family genetic history and information about personal habits were collected using a questionnaire survey. The patient’s medical history was unremarkable for infertility risk factors. Physical examination revealed normal penis and pubic hair. The patient’s endocrine status was assessed by electrochemiluminescent immunoassays for reproductive hormone levels including luteinizing hormone, follicle stimulating hormone, testosterone, and estradiol. The patient chose intracytoplasmic sperm injection (ICSI) as the treatment for his primary infertility. He agreed to participate in the research and signed informed consent. The present study was approved by the Ethics Committee of the First Hospital of Jilin University.

### Targeted exome sequencing

An in-house targeted gene panel (Beijing Medriv Academy of Genetics and Reproduction, Beijing, P. R. China), including *USP26*, was sequenced on the Illumina MiSeq platform (Illumina, San Diego, CA, U.S.A.). Selected targeted next-generation sequencing and data analysis were performed as described previously [15]. In brief, peripheral blood DNA samples were extracted using a DNA extraction kit (Beijing Tiangen Biotech Co., Ltd., China), and then the target gene panel was sequenced from the DNA sample using the Illumina MiSeq platform. The gene panel contained 52 genes (Table 1) involved in spermatogenesis failure, including *USP26*. After sequencing, low-quality base sequences and adaptor sequences were removed, and sequence alignment to the hg19 human reference sequence was performed using Burrows–Wheeler software. Duplicated reads from the library and PCR preparation were removed using Picard tools. Variants with minor allele frequencies greater than 1% in the databases, intronic variants, and synonymous variants were excluded. Nonsynonymous variants and splice site variants remained. The target gene mutation identifed was named by reference to the *USP26* gene sequence in the NCBI database (NM_031907.1). Mutation Taster (https://www.mutationtaster.org/) was used to assess the likely pathogenicity of the detected frameshift variation. The result was confirmed by conventional PCR and Sanger sequencing (ABI 3730XL, BGI Genomics, Shenzhen, China). Primers used for PCR of the detected mutation c.2195delT in *USP26* were 5′‐TGTAGGTCTTAGGAGGTTC‐3′(forward) and 5′‐CTCAGGAGATCGAGCAT‐3′(reverse).

**Table 1:** Lists of 52 genes tested in the study

USP26	AR	DNAH11	DNAH11	DNAH5
DNAI1	DNAI2	TEX11	ETV5	PLCZ1
CFTR	SPATA16	DPY19L2	AURKC	SOHLH2
SLC26A8	CATSPER1	SEPT12	NANOS1	CCDC39
RSPH1	RSPH4A	RSPH9	ZMYND10	DYX1C1
HYDIN	HEATR2	DNAAF1	DNAAF2	DNAAF3
SYCE1	SYCP3	SUN5	RHOXF1	RHOXF2
NR5A1	HSF2	KAL1	CHD7	PROK2
PROKR2	FGFR1	FGF8	KISS1R	NELF
WDR11	GNRHR	TAC3	TACR3	LEP
LEPR	ZMYDN15			

## Results

**Figure 1:**
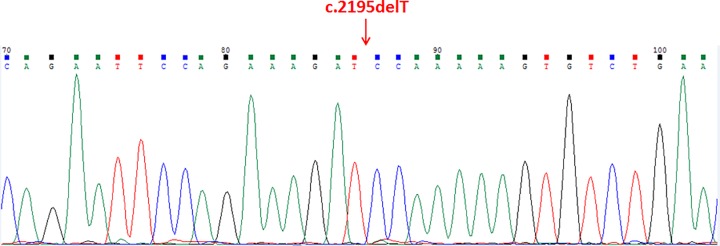
The *USP26* c.2195delT mutation was confirmed in our patient using Sanger sequencing The position was indicated by an red arrow.

**Figure 2:**
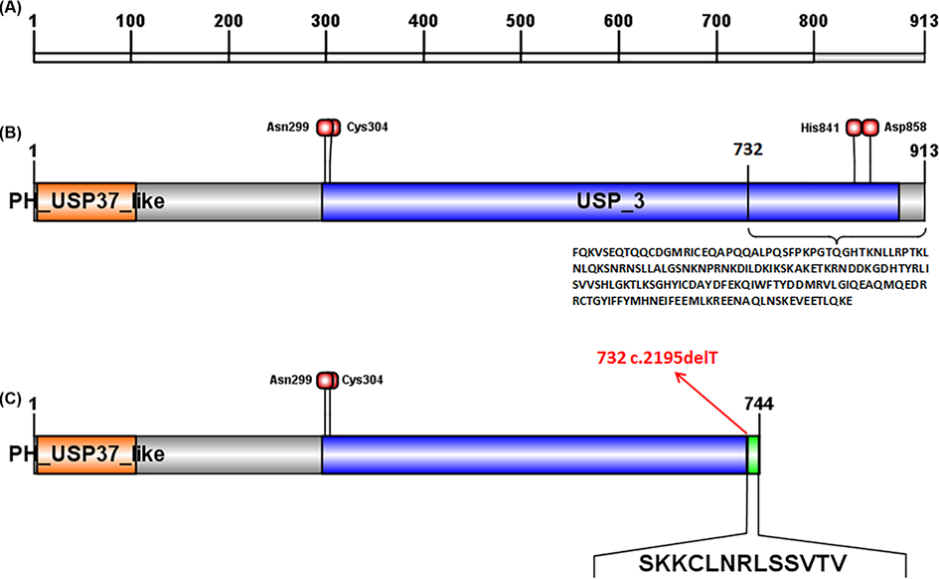
The normal and mutated USP26 domain structures (**A**) The amino acid positions are indicated above the rectangle. (**B**) The normal USP26 protein contains 913 amino acids. The orange box indicates the Pleckstrin homology-like domain of the Ubiquitin carboxyl-terminal hydrolase 37 (PH_USP37_like) domain, and the blue box indicates the USP_3 domain. The red square above represents the deubiquitinating enzyme active_site of Asn299, Cys304, His841, and Asp858. The sequence below the brace represents the normal sequence from amino acid residue 732 to 913. (**C**) The *USP26* mutation c.2195delT (red arrow) produces a truncated protein of 744 amino acids with loss of the active-site His841 and Asp858, which contain the conserved His- domain. This mutation leads to a frameshift from residue 732 (red arrow) to 744. The novel amino acids resulting from the frameshift are represented by the green box, and the mutation sequence is arranged below.

**Figure 3:**
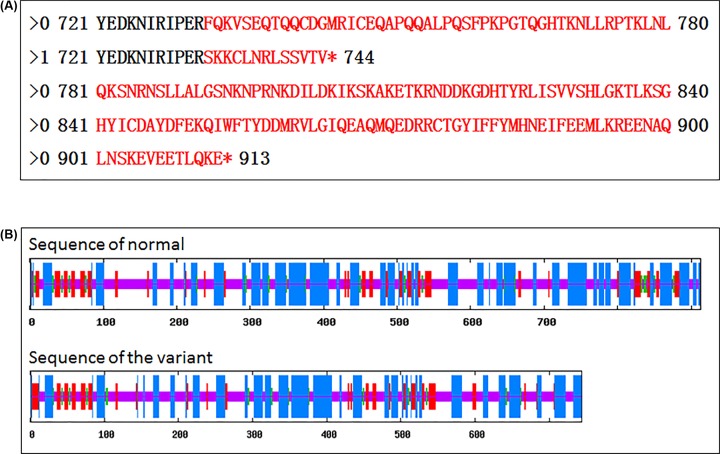
Amino acid sequence and protein secondary structure prediction results (**A**) “> 0” and “> 1” represent sequences from the normal and patient, respectively. Sequence alignment revealed a frameshit mutation from phenylalanine at codon 732 to the protein end (in red). This causes premature termination at codon 745. (**B**) The ratio of four main spatial structures in normal and variant sequences is depicted using blue, red, green, and purple lines representing alpha helix, extended strand, beta-turn, and random coil, respectively.

A novel frameshift mutation, c.2195delT (p.Phe732Serfs*14), not found in the ExAC or 1000G databases, was discovered in the patient (Figure 1). This c.2195delT mutation results in a frameshift at residue 732 and produces a truncated protein of 744 amino acids. This truncation results in the loss of the His841 and Asp858 active_site containing the conserved deubiquitinating enzyme His- domain. Mutation Taster predicted that this mutation was pathogenic. The truncated protein generated by the mutation might cause nonsense-mediated mRNA deday (NMD). Additionally, a locally installed third-party splice site prediction program, NNSplice from the Berkeley Drosophila Genome Project (http://fruitfly.org/seq_tools/splice.html) in Mutation Taster, predicted splice site changes in the identified mutation. The predicted effect of this loss-of-function mutation is shown in Figure 2. To predict how the mutation affected protein function and stability, the SOPMA (http:@www.ibcp.fr/predict.html) prediction tool was used. The amino acid sequences of the wild-type and the mutant are presented in Figure 3A. The SOPMA protein secondary structure prediction in normal and variant sequences were: alpha helix 39.76–36.69%, extended strand 11.94–12.50%, beta-turn 3.83–3.36%, and random coil 44.47–47.45% (Figure 3B).

The patient’s clinical data are listed in Table 2. This patient had normal hormone levels. The left and right testicular volumes were both 8 ml that is lower than that observed in normal men, suggesting testicular dysplasia. Semen analysis performed on two different occasions showed severe oligozoospermia. The sperm concentration was 2.16 mill./ml and the semen volume was 2.4 ml. Seminal plasma fructose, α-glycosidase, and zinc levels were all normal at 66.2, 40.1, and 7.9 μmol, respectively. The patient underwent one ICSI cycle. On the oocyte retrieval day, 11 MII oocytes were selected from 14 retrieved oocytes. Sperm collected from fresh semen were injected to the 11 MII oocytes. The cycle obtained a normal fertilization rate of 72.7% (8/11) and acquired four good quality embryos on day 3. Finally, two frozen thawed embryos scored 8I and 8II and were transferred into the uterus. The pregnancy resulted in the spontaneous delivery of a healthy male baby at 39 weeks gestation, weighing 3200 ***g*** and 50 cm long.

**Table 2:** Clinical and hormonal features and examination of semen in the patient

Characteristic	Measure	Normal range
Age (year)	30	
Height (cm)	178	
Weight (kg)	93	
Karyotype	46,XY	
AZF microdeletions	No deletions	
Left testicular volume (ml)	8	>10 ml
Right testicular volume (ml)	8	>10 ml
FSH (IU/l)	10.4	1.5–12.4 IU/l
LH (IU/l)	5.6	1.7–8.6 IU/l
Testosterone (nmol/l)	24.5	9.9–27.8 nmol/l
Estradiol (pmol/l)	82.7	27.96–155.92 pmol/l
Volume of semen (ml)	2.4	≥1.5 ml
pH of semen	7.2	≥7.2
Sperm count (mill./ml)	2.16	>15 ×10^6^/ml
Progressive sperm (%)	40.62	≥32%
Seminal plasma fructose (μmol)	66.2	≥13 μmol per ejaculation
Seminal plasma α-glycosidase (μmol)	40.1	≥20 μmol per ejaculation
Seminal plasma zinc (μmol)	7.9	≥2.4 μmol per ejaculation

## Discussion

DUBs regulate various cellular activities and the functions of various proteins by excising ubiquitin or ubiquitin-like proteins on the target protein [16]. They may influence spermatogenesis by regulating histone modification and protein turnover during meiosis [17]. *USP26*, a DUB family member, was first reported by Wang et al. [11] and is thought to be expressed only in testis. USPs have two short and well-conserved motifs in their catalytic domains called Cys and His boxes and contain residues critical for catalysis [18]. The *Wv/Wv* mouse model has no fertility and almost no germ cells. The expression of *USP26* in the testes of these mice is significantly lower than that in normal mice, suggesting the specific expression of *USP26* in germ cells [11]. RT-PCR was used to analyze *USP26* expression levels in male germ cells, and two expression phases were identified in different germ cells [19]. *USP26* is highly expressed in spermatogonia and pre-leptotene spermatocytes, absent in pachytene spermatocytes, and highly expressed in round spermatids. The testis-specific expression suggests that the *USP26* gene plays an important role in spermatogenesis, but its function remains unclear. In recent years, *USP26* has been reported to have an effect on AR. *USP26* encodes a nuclear protein that can be linked to AR to regulate AR deubiquitination levels. Indeed, AR signaling pathway abnormalities are closely related to spermatogenesis failure [20].

Previous studies on *USP26* have mostly focused on patients with azoospermia and oligozoospermia. Several *USP26* variations have been found in normal fertile men [12,21,22,23]. Particularly, a cluster 370-371insACA, 494T>C, and 1423C>T haplotype, resulting in an amino acid change of T123-124ins, L165S, and H475Y, respectively, is related to spermatogenesis failure of differing severities. This haplotype had a detection rate of 7.2% (8/111) in patients with Sertoli cell only syndrome [12] and 3% (6/200) in patients with non-obstructive azoospermia or severe oligospermia [23]. In the other two studies, the gene cluster haplotype was not detected in 232 patient groups, only one mutation was detected in 202 controls, and the semen quality of the positive carriers was normal [24,25].

Here, we performed sequencing of the entire *USP26* to identify previously-reported variations and to discover novel mutations in the gene. As a result, a novel frameshift mutation, c.2195delT (p.Phe732Serfs*14), was found in the patient and the most commonly reported *USP26* mutations (370-371insACA, 494T>C, and 1423C>T) were not detected. Liu et al. studied the enzymatic activity of 19 kinds of *USP26* variants to demonstrate the effect of mutations on male infertility. Compared with the wild-type, 18 mutations had no effect on enzyme activity, and the Q156H mutant was the only one in which enzyme activity disappeared [26]. Surprisingly, two terminal mutations (E174# and E189#) did not alter enzyme activity compared with the wild-type, which is inconsistent with the postulated theory. The reason for this may be that even if a termination mutation occurs, the coding process for the presence of the initiation codon in the following sequence would continue and enzyme activity would not be affected. The frameshift mutation from residue 732 in our study eventually led to premature termination, which was considered a pathogenic mutation. This mutation produces a truncated protein of 744 amino acids, and results in loss of the conserved deubiquitinating enzyme His- domain. Therefore, this mutation may be more capable of affecting protein function than a terminating mutation is. Using SOPMA, the truncated protein secondary structure was identified, and the specific gravity of the alpha helix and beta turn was shown to be reduced while that of the extended strand and random coil structure increased. This mutation induced a moderate change in the USP_3 domain structure of the truncated protein, which is critical for catalysis.

A previous report described two patients carrying a *USP26* missense mutation (R344W). This mutation was associated with NOA, and the patients had normal hormone levels, with FSH levels slightly higher in one patient [27]. The patient in our study showed severe oligozoospermia. His hormone levels were normal, but testicular volumes on both sides were significantly reduced, suggesting that he had testicular dysplasia. We hypothesized that this frameshift mutation may have an adverse effect on testicular development and affect testicular spermatogenesis. Using ICSI technology, the patient and his partner ultimately had a healthy male baby. Theoretically, this baby would not have inherited the X chromosome genetic defects of his father. We will continue to track the child’s growth and development, with a focus on fertility after adulthood.

In the present study, we reported a novel frameshift mutation, c.2195delT (p.Phe732Serfs*14), in *USP26* in a patient with severe oligozoospermia. Although we lacked a functional assay for this mutation, it was determined that the mutation resulted in a frameshift from residue 732 and produced a truncated protein of 744 amino acids, with loss of the the conserved deubiquitinating enzyme His- domain. Therefore, we speculate that this novel frameshift mutation may affect the structure and function of the USP26 protein to have an impact on spermatogenesis. Our results will enrich the *USP26* mutation database and provide a genetic basis for the etiological diagnosis of male infertility.

## Abbreviations

AR: androgen receptor
DUB: deubiquitinating enzymes
E2: estradiol
FSH: follicle stimulating hormone
ICSI: intracytoplasmic sperm injection
LH: luteinizing hormone
T: testosterone
USP26: ubiquitin-specific protease 26
